# Impact of Daily versus Weekly Supply of Locally Produced Ready-to-Use Food on Growth of Moderately Wasted Children on Nias Island, Indonesia

**DOI:** 10.5402/2013/412145

**Published:** 2013-02-27

**Authors:** Ratna Chrismiari Purwestri, Veronika Scherbaum, Dyah Ayu Inayati, Nia Novita Wirawan, Julia Suryantan, Maurice Alexander Bloem, Rosnani Verba Pangaribuan, Wolfgang Stuetz, Volker Hoffmann, Matin Qaim, Hans Konrad Biesalski, Anne Camilla Bellows

**Affiliations:** ^1^Department of Gender and Nutrition, Institute of Social Sciences in Agriculture, University of Hohenheim and Center of Gender and Nutrition, Schloss, Museumfluegel, 70593 Stuttgart, Germany; ^2^Institute for Biological Chemistry and Nutrition, University of Hohenheim, 70593 Stuttgart, Germany; ^3^Study Program Nutrition, Faculty of Medicine, University of Brawijaya, Malang 65145, Indonesia; ^4^Church World Service, Jakarta 12560, Indonesia; ^5^Church World Service, New York, NY 10115, USA; ^6^SEAMEO TROPMED Regional Centre for Community Nutrition, University of Indonesia, Jakarta 10430, Indonesia; ^7^Institute of Nutrition, Friedrich-Schiller-University of Jena, 07743 Jena, Germany; ^8^Department of Agricultural Economics and Rural Development, University of Goettingen, 37073 Goettingen, Germany; ^9^Department of Public Health, Food Studies, and Nutrition, David B. Falk College, Syracuse University, NY 13244, USA

## Abstract

This study reports the outcomes of daily (semi-urban areas) and weekly (remote rural regions) programs for moderately wasted children supplemented with locally produced ready-to-use foods in the form of fortified cereal/nut/legume-based biscuits on Nias Island, Indonesia (RUF-Nias biscuit). Thirty-four children in daily and twenty children in weekly programs aged ≥6 to <60 months with weight-for-height *z*-score (WHZ) ≥ −3 to < −2 SD were recruited (October 2007–June 2008) on Nias and admitted into existing nutrition centers in the Church World Service project area. Individual discharge criterion was WHZ ≥ −1.5 SD. Weight gain of the children in daily and weekly programs was 3.9 ± 3.8 and 2.0 ± 2.0 g/kg/day, respectively. A higher proportion of children in daily than weekly programs reached target WHZ (76% vs. 35%, *P* = 0.004). Weight gain at program discharge/closure was highly predicted (*R* ^2^ = 0.228, *P* < 0.001) by compliance to RUF biscuits: high vs. low compliance resulted in a 1.33 (95% CI 0.16 to 1.53) g/kg/day higher weight gain. Compliance and admission in daily programs were significant factors in reducing the risk of not reaching the discharge criterion. However, mothers complained more frequently about time constraints in the daily relative to weekly programs.

## 1. Introduction

 Globally, about 36 million children are suffering from moderate acute malnutrition (MAM) [1]. These children are usually supplemented with uncooked food supplements such as corn soy blend, often substituted with sugar and oil [2]. In 2008, a WHO/UNICEF/WFP/UNHCR meeting focused on management of MAM children and highlighted the importance of dietary management by providing both nutritional counseling and locally adapted food supplements [3, 4]. To date, fortified peanut/milk paste ready-to-use therapeutic foods (RUTFs), which are mainly commercially produced at international or national levels, have been tested with promising weight gain for severely, moderately, and mildly wasted children in the community-based settings [5–9]. More recently, alternative ready-to-use foods (RUFs) in the form of fortified cereal/nut/legume-based biscuits were designed at the University of Hohenheim, Germany, for severe acute malnutrition (SAM) among children. Out of nine RUF recipes, two were locally selected, produced at village level, and tested for the children suffering from moderate forms of wasting on Nias Island, Indonesia, within daily (in semi-urban areas) and weekly supervision and distribution programs (in rural remote regions) [10]. This study reports comparison between daily and weekly program outcomes of moderately wasted children whose diets were supplemented with locally produced RUF-Nias biscuits. 

## 2. Methods

### 2.1. Population under Study

The study was part of a research project that aimed to compare different intervention programs to prevent and rehabilitate mildly wasted (results presented elsewhere [11]) and moderately wasted children in the Church World Service (CWS) project area on Nias Island, Indonesia, from October 2007 to June 2008. During the field work period, the children were recruited from the preexisting community-based screening programs in the CWS project area. Inclusion criteria of the children were weight-for-height *z*-score (WHZ) ≥ −3 to < −2 SD according to WHO reference data [12], aged ≥6 months to < 60 months old, and no birth defect or disease that could limit the ad libitum food intake. Eligible children in semi-urban areas were allocated to daily programs, while those in rural remote regions were assigned to weekly programs (no randomization). Individual discharge criterion was WHZ ≥ −1.5 SD. 

To detect a weight increment difference of 2.1 ± 2.4 g/kg body weight/day (g/kg/day) [5], with a confidence level of 95% and a power of 0.8, a minimum sample size of 20 children per program was calculated [13, 14]. In this study, 34 children were appointed to the daily (semi-urban settings) and 20 to the weekly distribution and supervision program settings (in rural areas), respectively.

Informed consent was obtained from all mothers/caregivers of admitted children. The study conformed to the provisions of the Declaration of Helsinki and was approved by the Ethics Committee of the Faculty of Medicine, Brawijaya University, Malang, Indonesia (no. 25/PEPK/VIII/2007). 

### 2.2. Design of the Study

**Figure 1:**
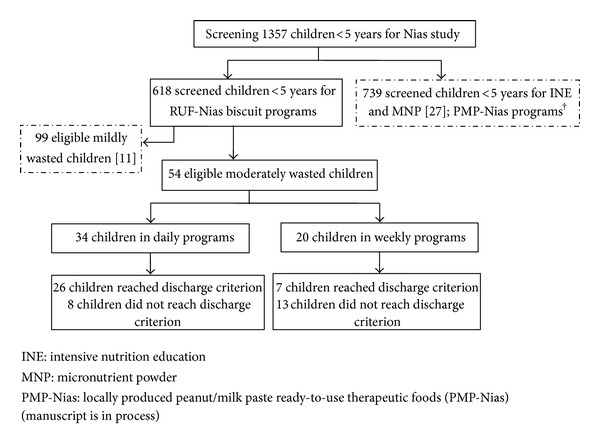
Study profile of children receiving RUF-Nias biscuits in daily and weekly programs (see [11, 27]).

Screening of children was ongoing on a monthly basis to identify new cases of wasted children. In total, 618 children were screened for the RUF-Nias biscuits intervention study. A total of 99 of these children were mildly wasted [11], and 54 of them were moderately wasted. Of the moderately wasted children, 34 and 20 children were allocated to daily (semi-urban settings) and weekly programs (in rural remote regions), respectively (Figure 1). A longitudinal nonrandomized food-based intervention study was applied in the preexisting nutrition centers (at village level) in CWS project area. In each nutrition center, RUF-Nias biscuits were produced by voluntary workers, CWS field officers, and motivated mothers/caregivers once a week.

In daily programs, each eligible child (index child) was provided with a portion of RUF-Nias biscuits every day (except Sunday), and about one-third of which was to be eaten within a supervised feeding setting in the nutrition center. Compliance with the take-home ration of RUF-Nias biscuits and morbidity were routinely assessed the following day at the nutrition center. The index children were weighed 2-3 times per week; height was measured once per month. In weekly programs, however, all basic activities were performed only once per week and mothers/caregivers received take-home portions for the index children for each of the other days of the week, with the advice to offer it daily between meals. Compliance with RUF-Nias biscuits consumption could only be observed and monitored once a week. WHZ, height-for-age *z*-score (HAZ), and mid-upper arm circumference (MUAC) were collected for each index child at program admission, during the intervention period, and discharge/closure. The weight of the index child was assessed using a hanging spring scale; height was measured using a height/length board for children above/below 2 years of age; MUAC was determined by using MUAC inserting tape (Ministry of Health, Indonesia). General background information (e.g., age and sex) and a 24-hour dietary intake recall of the index children during admission time, as well as socio-economic characteristics of their families, were provided by the mothers/caregivers on the basis of a standardized questionnaire. 

Length of stay for the children who reached discharge criterion (RDC) was defined as number of days until WHZ ≥ −1.5 SD was reached, while length of stay for non-RDC children included the time from admission until program closure. Defaulter was defined for children who were absent in at least two consecutive scheduled daily/weekly program activities, who were home visited at least two times because they were absent in the program activities, and whose mothers/caregivers withdrew from the program. Additionally, field-note observations and interviews with seven non-RDC caregivers (four and three caregivers from daily and weekly programs, resp.), who were willing to participate in in-depth interviews for about 60 to 80 minutes, were carried out to understand the underlying causes and culturally determined reasons for not reaching discharge criterion during the program period. 

### 2.3. Locally Produced RUF-Nias Biscuits

Nine different recipes of RUF biscuits were developed at University of Hohenheim, Germany. After organoleptic and sensory evaluation for appearance, color, texture, smell, shelf life, and taste under given local climate conditions, two out of nine recipes (soybean based and mungbean based) were selected and locally produced for this intervention study [10]. Ingredients of the locally produced RUF-Nias biscuits were wheat flour, peanut flour, refined sugar, palm oil, egg yolk and white, soybean or mungbean flour, and micronutrient powder donated from DSM Nutritional Product Ltd., Basel (DSM). Flour from soybean/mungbean was used interchangeably and was roasted prior to biscuit production to reduce the phytate content and to improve bioavailability of antioxidants and iron [15–17].

Originally, the RUF-Nias biscuits were developed for rehabilitation of children suffering from severe acute malnutrition (SAM) [10, 18], and therefore the amounts of macro- and micronutrients are comparable to the level of proposed intake for children suffering from SAM (see Table 1).

**Figure 2:**
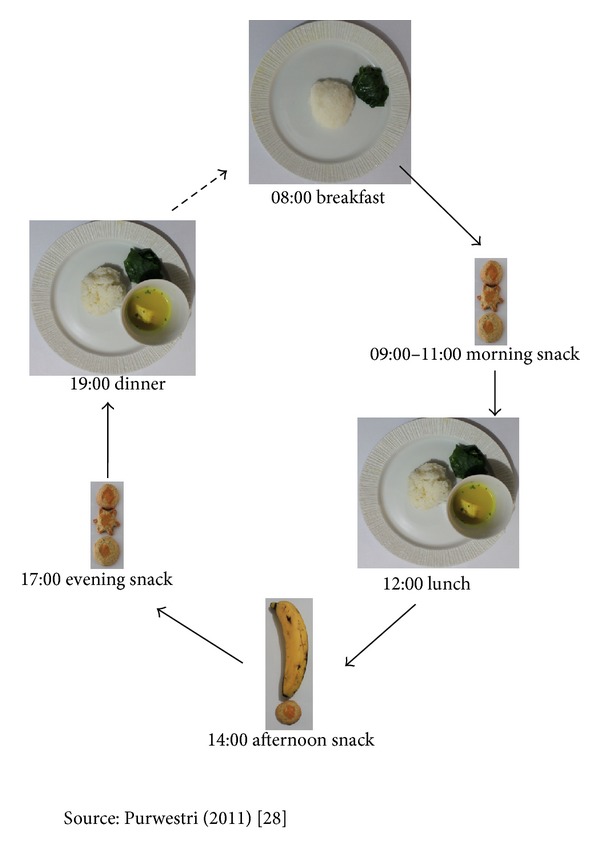
Example of feeding recommendation of RUF-Nias biscuits for non-breastfed children (aged ≥ 24 to < 36 months) based on average 24-hour dietary recall (consisted of rice, cassava leaves, and fish soup) (see [28]).

Children in daily and weekly programs received a calculated RUF portion per day based on the weight of the individual child, to cover about 60% of the recommended daily energy requirements, as per Indonesian guidelines [19]. About 10% was added to the original estimation of ±50% daily energy requirement fulfillment for moderately wasted children [8, 20] in order to compensate for the possibility of the RUF-Nias biscuits being shared with other family members [10]. In addition, we stressed that RUF-Nias biscuits should be considered as a “medicine” for the index children and consumed in-between meals (see Figure 2). 

### 2.4. Data Analysis

Continuous data was first checked for normal distribution using quintile-quintile plots of normality. All nutritional indicators, which were found to be skewed, were log transformed in order to reach normal distribution for further analyses. For allowing comparison with other published studies, the outcomes in nutritional indicators were presented as mean ±SD. Differences in continuous variables, such as weight, height, WHZ, HAZ, and MUAC between daily and weekly programs were analyzed using independent *t*-test, while Fisher's exact test was used to analyze differences in proportions between daily and weekly programs and RDC versus non-RDC. Multivariate logistic and linear regression analyses with a forward stepwise approach were applied to identify independent risk factors of not reaching discharge criterion and predictors of the children's weight gain at program discharge/closure, respectively. The following covariates were assessed: children's age and WHZ at admission, compliance on RUF consumption, type of program, and morbidity of the children during the program period. Statistical analyses were performed using PASW/SPSS version 18.0 for Windows software packages (SPSS Inc., Chicago, IL, USA). 

**Figure 3:**
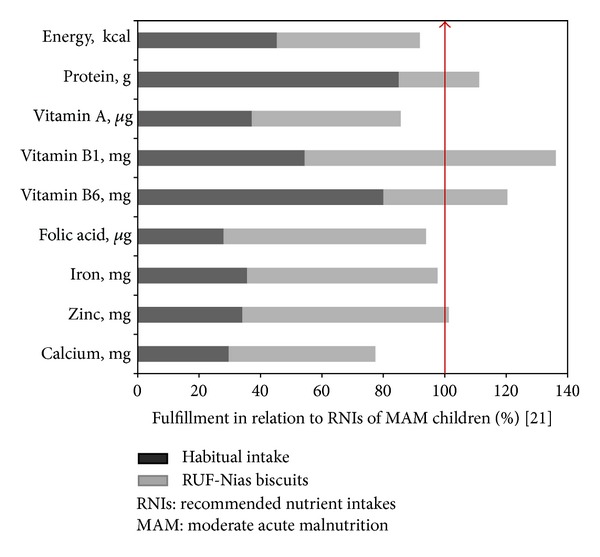
Selected macro- and micronutrients of mean habitual dietary intake and RUF-Nias biscuits of moderately wasted children in comparison to RNIs for MAM children (for suggested nutrient density that should be achieved in the diet when specially fortified supplementary foods are used in the program to treat moderately wasted children) [21].

Data on weight and height were transformed to *z*-scores of WHZ and HAZ according to WHO reference data 2005 [12] using Emergency Nutrition Assessment (ENA for SMART) version 2007. Data on food intake were analyzed using Nutrisurvey version 2007. As shown in Figure 3, mean selected nutrients from habitual intake of the index children (based on a 24-hour recall at admission) together with average consumption of RUF-Nias biscuits (collected during program period) were compared in relation to the dietary guidelines for MAM children [21].

## 3. Results

All children admitted into daily or weekly RUF-Nias biscuit intervention programs had similar age and anthropometric indicators (weight, height, WHZ, HAZ, and MUAC). Program outcomes between girls and boys did not differ significantly (*P* = 0.260) in daily (41% girls) and weekly (60% girls) programs.

Mean age, education, and occupation of the mothers are shown in Table 2. In daily programs, women were significantly younger, less likely to work as farmers and had nearly the same education level as women in weekly programs. A very high proportion of respondents in daily and weekly programs (91.2 versus 100%, *P* = 0.287) came from families with a total household income lower than US$ 1.25/day of Purchasing Parity Power (PPP) [22] per family member. 

Moderately wasted children in the daily and weekly programs had similar inadequate habitual dietary intake prior to admission (Table 3). Average percent fulfillment of selected macro- and micronutrient intake at baseline was considerably below dietary recommendations for well-nourished Indonesian children [19]. The majority of the children's mean habitual nutrient intake, together with the calculated amount of RUF-Nias biscuits, was approaching the 2009 published recommended levels for MAM children [21] (Figure 3). 

As shown in Table 4, weight gain at program discharge/closure of children assigned in daily and weekly programs was 3.9 g/kg/day and 2.0 g/kg/day (*P* = 0.117), respectively. WHZ score at discharge/closure was significantly higher in daily than in weekly programs (*P* = 0.027). Furthermore, the proportion of children who reached discharge criterion of WHZ ≥ −1.5 within a similar average length of stay (7–9 weeks) was significantly higher in daily than in weekly programs (76.5% versus 35%, *P* = 0.004). 

Weight gain (in g/kg/day) of children at program discharge/closure was highly predicted by high compliance (*R* ^2^ = 0.228, *P* < 0.001). Children with a high compliance (≥80% consumption of prescribed RUF biscuits) had a higher mean daily weight gain of 1.33 (95% CI 1.16 to 1.53) g/kg body weight than children with low compliance (<80% RUF biscuit consumption). Logistic regression analysis revealed low compliance and weekly versus daily program as independent risk factors of not reaching discharge criterion; children in the low compliant versus high compliant group had a 40- (95% CI 5 to 320) fold and children in the weekly versus the daily program had a 17- (95% CI 3 to 92) fold higher risk of not reaching the discharge criterion (both *P* < 0.001). Children's age and WHZ at admission and morbidity during program periodwere not associated with the risk of not reaching discharge criterion in this study setting.

In Table 5, a pooled dataset of children who reached discharge criterion (RDC) versus non-RDC is presented showing that the nutritional indicators of RDC and non-RDC children in daily and weekly programs were not significantly different. Children who reached discharge criterion (WHZ ≥ −1.5 SD) showed significantly better improvements in weight, WHZ, and MUAC than non-RDC children. In both programs, poor compliance (consumption of <80% of the daily allotted portion of RUF-Nias biscuits) was most likely in non-RDC children (±50%) compared to RDC children (±6%).

## 4. Discussion

Based on a 24-hour dietary recall, mean habitual nutrient intake of the moderately wasted children was considerably lower than the dietary guidelines for Indonesian well-nourished children (see Table 3). Together with the fortified RUF-Nias biscuits, about two-thirds of the recommended levels of micronutrients were reached, while energy, Vitamin B1, and protein reached or even exceeded the RNIs for MAM children [21] (see Figure 3). Average habitual protein intake of the moderately wasted children was relatively high because of the daily fish consumption (e.g., sardines and anchovies) on Nias Island. 

Moderately wasted children who consumed about 100 g (±500 kcal) RUF-Nias biscuits in the daily programs gained 3.9 ± 3.8 g/kg/day in about 51 days, whereas weight gain of those in the less-supervised weekly programs was 2.0 ± 2.0 g/kg/day within approximately 62 days. The proportion of children who reached WHZ ≥ −1.5 SD (RDC) in daily programs was 76.5%. In contrast, the proportion of RDC children was significantly lower in less-supervised weekly programs (35.0%). No child defaulted from either program.

Monitoring results of the children's diseases during the program period suggested that, in most cases, frequent illnesses were not the main cause of lack for appropriate weight gain of moderately wasted children in our study area. The intervention program referred sick children to local health centers. However, the cost of medicine and geographic settings in combination with limitations of local health systems seemed to constrain them from seeking early medication. This situation was more profound among the children in the weekly programs who lived in rural remote regions that linked with challenging infrastructure and poor public/health facilities, which could contribute to the lower proportion of RDC children in the weekly than in the daily programs.

Prior to this study, CWS had already established nutrition intervention programs like cooking demonstrations at its diverse sites. Geographical differences (more urban/accessible versus more rural/less accessible) largely influenced the regularity of program delivery (daily versus weekly, resp.). We note that randomization of nutrition centers could not be applied because, in the more urban (i.e., semi-urban) areas, they were established too close together and it was difficult to avoid exchange of information, as well as possible jealousy that could arise over more or less intensive supervision in a neighboring program. With regard to the more remote rural research sites, houses were scattered and often far away from the nutrition center; daily supervision was impractical.

Based on results of in-depth interviews among mothers of non-RDC children who were willing to be interviewed, children preferred snacks bought from nearby shops (e.g., candies, chocolates, wafers, biscuits, and jellies) as compared to the distributed RUF-Nias biscuits because the latter was perceived as “boring” in terms of taste and appearance. Mothers of children who did not reach discharge criterion also mentioned that illnesses were considered among the reasons for not reaching the criterion. A few lower income caregivers, as well as lower educated mothers and grandmothers, tended toward noncompliance with instructions for giving RUF-Nias biscuits.

Additionally, we observed that all voluntary workers and all caregivers in the Nias project were female and that they lived in a patrilineal and patriarchal social system [23, 24]. About 47–60% of the respondents lived together with the husband's extended family in one house (see Table 2). Other families lived in separate houses that were located close to the houses of the husband's parents. As mentioned above, these living arrangements intensify the strong influence of the husband's family and most particularly that of the paternal grandmother. On average, the majority of women in the study area were engaged in income generating activities outside the home and spent only about two hours in direct child care [25]. Therefore, child feeding practices at home and the type/variety of foods given are greatly influenced by grandmothers, particularly on the paternal side. Although longer travelling time to a nutrition center meant that rural mothers/caregivers had to leave their households and farming activities for a longer period, we observed that they were nevertheless very willing to attend the nutrition center's activities. With consideration for the additional time involved, caregivers in semi-urban areas regularly complained about time constraints created by program involvement.

## 5. Limitations and Outlook

Prior to this intervention study, nutrition-related teaching activities had already been introduced in every village where more than 6 wasted children were found [26]. We found that village randomization was not feasible in the semi-urban areas because the existing nutrition centers were located too close to each other, and on the other hand a daily program was impractical in rural areas. Therefore, we recommend performing a similar intervention study in larger regions by randomizing semi-urban nutrition centers to daily and weekly RUF supplements for comparison of the mode of service delivery. Our results also confirmed that a weekly program was feasible in a rural remote region and produced a satisfactory program outcome.

Besides provision of locally produced RUF-Nias biscuits, we also administered locally produced peanut/milk paste (PMP-Nias) with similar macro- and micronutrient contents [10] to eight moderately and thirty-seven mildly wasted children in another region (Figure 1) on Nias Island. Of eight moderately wasted children, five did not reach discharge criterion of WHZ ≥ −1.5 SD, two defaulted from the programs, and only one child recovered fully. Generally, we observed that RUF-Nias biscuits were highly accepted by both the caregivers and the concerned children in contrast to peanut/milk paste mainly because children were not accustomed to its taste and parents did not like the recipe (will be published elsewhere). 

Compliance with RUF-Nias biscuits consumption should be enhanced by altering the recipe through inclusion of different types of local foods rich in micronutrients (e.g., local seeds, spices, and milk or fish powder) that were already included in the original nine recipes of RUFs [10]. Furthermore, frequent home visits for children with poor compliance records and inclusion of other family members, especially paternal grandmothers, in behavioral change communication sessions are also recommended to improve the compliance on RUF-biscuits consumption.

## 6. Conclusion and Recommendation

Locally produced RUF-Nias biscuits in the form of in-between snacks resulted in promising weight gain in moderately wasted children on Nias Island, Indonesia. Provision of locally produced RUF biscuits resulted in a significant weight gain in moderately wasted children and could be an alternative approach to the commercially produced RUF. Further studies are needed to identify ways in which compliance of RUF consumption could be improved in both daily and weekly programs. 

## Figures and Tables

**Table 1:** Macro- and micronutrients in 100 g RUF-Nias biscuits^†^.

Micronutrients	Soybean-based RUF-Nias + premix [10]	Mungbean-based RUF-Nias + premix [10]
Macronutrients		
Energy, kcal	536	523
Protein, %	10	8
Fat, %	58	60
Micronutrients		
Vitamin A, *μ*g	979.0	970.8
Vitamin D, *μ*g	16.5	16.4
Vitamin C, mg	54.3	54.0
Thiamine B1, mg	0.9	0.9
Riboflavin, mg	1.9	1.9
Vitamin B6, mg	0.7	0.7
Vitamin B12, *µ*g	1.9	1.9
Niacin, mg	11.7	11.2
Biotin, *μ*g	66.0	65.5
Folic acid, *μ*g	255.8	257.2
Vitamin K, *μ*g	28.3	27.2
Vitamin E, mg	20.1	19.4
Pantothenate, mg	4.0	3.9
Calcium, mg	345.9	320.2
Iron, mg	13.9	12.6
Iodine, *μ*g	120.8	120.7
Zinc, mg	14.9	14.4
Sodium, mg	16.0	14.8
Potassium, mg	953.9	803.4
Magnesium, mg	91.6	67.0
Phosphorus, mg	350.7	286.5
Copper, mg	2.3	2.1
Selenium, *μ*g	30.6	30.6

^†^Level of micronutrients in the premix was provided by DSM.

**Table 2:** Selected characteristics of the eligible moderately wasted children and their families during admission, by daily and weekly programs^†^.

Indicators	Daily programs	Weekly programs	*P* value*
*N*	34	20	
Characteristics of the children			
Female children	41.2 (14)	60.0 (12)	0.260
Age of children, months	30.3 ± 12.8	30.8 ± 15.7	0.900
Children still breastfed	47.1 (16)	35.0 (7)	0.412
Weight, kg	9.3 ± 1.7	9.1 ± 2.1	0.761
Height/length, cm	83.3 ± 8.5	83.1 ± 10.6	0.962
WHZ	−2.35 ± 0.27	−2.48 ± 0.25	0.079
HAZ	−2.12 ± 1.12	−2.14 ± 0.95	0.939
MUAC, cm	13.1 ± 1.0	13.0 ± 0.8	0.886
Characteristics of the families			
Age of parents, years			
Mothers	27.7 ± 4.2	30.7 ± 4.8	0.019
Fathers	30.7 ± 6.2	38.7 ± 8.7	0.002
Occupation of parents (farmer)			
Mothers	67.6 (23)	85.0 (17)	0.208
Fathers	47.1 (16)	75.0 (15)	0.053
Education of caregivers (≥6 years of schooling)			
Maternal education	38.2 (13)	40.0 (8)	1.000
Father education	76.4 (26)	60.0 (12)	0.230
Lived with extended family	47.1 (16)	60.0 (12)	0.408
Income per capita per day^‡^			
<US$1.25/day	91.2 (31)	100.0 (20)	0.287

WHZ: weight-for-height *z*-score; HAZ: height-for-age *z*-score; MUAC: mid-upper arm circumference.

^†^Continuous variables written as mean ± SD, categorical variables presented as % (*n*).

^‡^Income data was derived from average cash money earned every month and did not include household valuable assets, agriculture production, savings, or aids. US$1 equal to ±Rp 9,230 using currency rates in 2007, US$1.25 PPP equal to ±Rp 4,918 [22].

*Independent *t*-test (continuous data) or Fisher's exact test (percentages) for comparing daily and weekly programs.

**Table 3:** Averages of selected nutrients in habitual intake of moderately wasted children (aged ≥6 to <60 months) and percent fulfillment according to Indonesian dietary recommendations for well-nourished children at program admission, by daily and weekly programs^†^.

Nutrients	Daily programs (*N* = 34)		Weekly programs (*N* = 20)		*P* value^‡^	
	Mean intake	% fulfillment	Mean intake	% fulfillment	Mean intake	% fulfillment
Energy, kcal	515.0 ± 355.5	45.2 ± 29.4	519.4 ± 236.7	45.6 ± 24.0	0.962	0.959
Protein, g	24.8 ± 21.7	85.8 ± 71.4	24.0 ± 18.7	83.7 ± 64.2	0.890	0.915
Vitamin A, *μ*g	179.8 ± 216.0	43.7 ± 53.4	108.4 ± 89.3	26.2 ± 22.1	0.096	0.100
Vitamin B1, mg	0.3 ± 0.3	58.3 ± 55.5	0.3 ± 0.2	47.8 ± 39.4	0.380	0.462
Vitamin B6, mg	0.4 ± 0.3	82.1 ± 39.4	0.4 ± 0.2	65.5 ± 43.4	0.268	0.255
Folic acid, *μ*g	53.8 ± 53.5	33.2 ± 34.2	31.4 ± 34.3	19.1 ± 22.8	0.100	0.109
Iron, mg	3.2 ± 3.7	38.8 ± 45.8	2.5 ± 2.3	30.2 ± 28.4	0.475	0.457
Zinc, mg	2.5 ± 2.6	37.1 ± 44.7	1.8 ± 1.1	29.1 ± 27.7	0.297	0.477
Calcium, mg	174.0 ± 315.9	25.8 ± 39.6	72.2 ± 70.2	36.4 ± 83.8	0.163	0.532

^†^Continuous variables written as mean ± SD.

^‡^Independent *t*-test for comparing daily and weekly programs.

**Table 4:** Anthropometric indices and other program outcomes (during admission and before discharge/program closure) of moderately wasted children, by daily and weekly programs^†^.

Indicators	Daily programs	Weekly programs	*P* value^††^
*N*	34	20	
Weight			
Admission, kg	9.3 ± 1.7	9.1 ± 2.1	0.761
Discharge, kg	10.2 ± 1.9	9.9 ± 2.2	0.572
Difference, kg	+0.9 ± 0.8	+0.8 ± 0.6	0.778
Weight gain, kg/day	+0.04 ± 0.04	+0.03 ± 0.06	0.600
Weight gain, g/kg/day	+3.9 ± 3.8	+2.0 ± 2.0	0.117
Height			
Admission, cm	83.3 ± 8.5	83.1 ± 10.6	0.962
Discharge, cm	84.5 ± 8.7	85.0 ± 10.7	0.857
Difference, cm	+1.2 ± 1.9	+1.8 ± 1.9	0.945
WHZ			
Admission	−2.35 ± 0.27	−2.48 ± 0.25	0.079
Discharge	−1.44 ± 0.72	−1.93 ± 0.84	0.027
Change in *z*-score	+0.9 ± 0.8	+0.6 ± 0.8	0.270
HAZ			
Admission	−2.12 ± 1.12	−2.14 ± 0.95	0.939
Discharge	−2.07 ± 0.98	−2.15 ± 1.13	0.803
Change in *z*-score	0.0 ± 0.6	0.0 ± 0.4	0.958
MUAC			
Admission, cm	13.1 ± 1.0	13.0 ± 0.8	0.886
Discharge, cm	13.8 ± 0.9	13.4 ± 0.9	0.176
Difference, cm	+0.7 ± 0.9	+0.4 ± 0.6	0.230
Children who reached discharge criterion, % (*n*)	76.5 (26)	35.0 (7)	0.004
Length of stay^‡^, day	51.2 ± 40.0	61.8 ± 35.1	0.665
RUF intake, g/day	83.3 ± 32.6	78.4 ± 33.0	0.594
Poor compliance*, % (*n*)	23.5 (8)	20.0 (4)	1.000
Prevalence of illnesses at admission, % (*n*)			
Diarrhea	5.9 (2)	15.0 (3)	0.369
Respiratory infection	61.8 (21)	55.0 (11)	0.389
Fever	41.2 (14)	30.0 (6)	0.381
Number of children with illnesses during program period, % (*n*)			
Diarrhea	11.8 (4)	20.0 (4)	0.450
Respiratory infection	58.8 (20)	35.0 (7)	0.775
Fever	11.8 (14)	20.0 (6)	0.450

WHZ: weight-for-height *Z*-score; HAZ: height-for-age *Z*-score; MUAC: mid-upper arm circumference, RUF: ready-to-use foods.

^†^Continuous variables written as mean ± SD, categorical variables presented as % (*n*).

^‡^Length of stay for children who reached discharge criterion was defined as number of days until reaching WHZ ≥−1.5 SD; length of stay for children who did not reach discharge criterion was defined as number of days until program closure.

*Poor compliance was defined as reported inadequate RUF-Nias biscuit consumption (<80%) during the program period.

^††^Independent *t*-test (continuous data) or Fisher's exact test (percentages) for comparing daily and weekly programs.

**Table 5:** Selected characteristics and outcomes of pooled data of RDC and non-RDC children^†^.

Outcomes	RDC children	Non-RDC children	*P* value^††^
*N*	33	21	
Weight			
Admission, kg	9.2 ± 1.7	9.2 ± 2.1	0.905
Discharge, kg	10.4 ± 2.0	9.6 ± 2.0	0.131
Difference, kg	+1.2 ± 0.6	+0.4 ± 0.7	0.000
Weight gain, kg/day	+0.05 ± 0.05	0.01 ± 0.01	0.000
Weight gain, g/kg/day	+4.8 ± 3.3	+0.6 ± 0.9	0.000
Height			
Admission, cm	83.2 ± 9.0	83.2 ± 10.5	0.974
Discharge, cm	84.3 ± 9.0	85.3 ± 10.3	0.705
Difference, cm	+1.0 ± 1.7	+2.1 ± 2.0	0.286
WHZ			
Admission	−2.37 ± 0.28	−2.44 ± 0.25	0.318
Discharge	−1.11 ± 0.26	−2.42 ± 0.69	0.000
Change in *z*-score	+1.25 ± 0.41	+0.20 ± 0.67	0.000
HAZ			
Admission	−2.14 ± 1.04	−2.11 ± 1.09	0.909
Discharge	−2.16 ± 1.02	−2.02 ± 1.06	0.631
Change in *z*-score	−0.02 ± 0.46	+0.09 ± 0.68	0.572
MUAC			
Admission, cm	13.1 ± 1.0	12.9 ± 0.8	0.372
Discharge, cm	14.0 ± 0.8	13.2 ± 0.8	0.002
Difference, cm	+0.8 ± 0.9	+0.3 ± 0.5	0.011
Length of stay^‡^, day	39.5 ± 33.0	73.8 ± 33.2	0.000
RUF intake, g/day	89.8 ± 31.0	68.4 ± 31.9	0.017
Poor compliance*, % (*n*)	6.3 (2)	47.6 (10)	0.001

RDC: reached discharge criterion; WHZ: weight-for-height *Z*-score; HAZ: height-for-age *Z*-score; MUAC: mid-upper arm circumference, RUF: ready-to-use foods.

^†^Continuous variables written as mean ± SD, categorical variables presented as % (*n*).

^‡^Length of stay for children who reach discharge criterion was defined as number of days until reaching WHZ ≥−1.5 SD; length of stay for children who did not reach discharge criterion was defined as number of days until program closure.

*Poor compliance was defined as reported inadequate RUF-Nias biscuit consumption (<80%) during the program period.

^††^Independent *t*-test (continuous data) or Fisher's exact test (percentages) for comparing RDC versus non-RDC children.
